# Magnesium Attenuates Phosphate-Induced Deregulation of a MicroRNA Signature and Prevents Modulation of Smad1 and Osterix during the Course of Vascular Calcification

**DOI:** 10.1155/2016/7419524

**Published:** 2016-06-22

**Authors:** Loïc Louvet, Laurent Metzinger, Janine Büchel, Sonja Steppan, Ziad A. Massy

**Affiliations:** ^1^INSERM U 1088, CURS, University of Picardie Jules Verne, Amiens, France; ^2^Fresenius Medical Care Deutschland GmbH, Bad Homburg, Germany; ^3^Division of Nephrology, Ambroise Paré University Hospital, APHP, University of Paris Ouest-Versailles-St-Quentin-en-Yvelines (UVSQ), Boulogne-Billancourt, Paris, France; ^4^INSERM U 1018, Research Centre in Epidemiology and Population Health (CESP) Team 5, Villejuif, France

## Abstract

Vascular calcification (VC) is prevalent in patients suffering from chronic kidney disease (CKD). High phosphate levels promote VC by inducing abnormalities in mineral and bone metabolism. Previously, we demonstrated that magnesium (Mg^2+^) prevents inorganic phosphate- (Pi-) induced VC in human aortic vascular smooth muscle cells (HAVSMC). As microRNAs (miR) modulate gene expression, we investigated the role of miR-29b, -30b, -125b, -133a, -143, and -204 in the protective effect of Mg^2+^ on VC. HAVSMC were cultured in the presence of 3 mM Pi with or without 2 mM Mg^2+^ chloride. Total RNA was extracted after 4 h, 24 h, day 3, day 7, and day 10. miR-30b, -133a, and -143 were downregulated during the time course of Pi-induced VC, whereas the addition of Mg^2+^ restored (miR-30b) or improved (miR-133a, miR-143) their expression. The expression of specific targets Smad1 and Osterix was significantly increased in the presence of Pi and restored by coincubation with Mg^2+^. As miR-30b, miR-133a, and miR-143 are negatively regulated by Pi and restored by Mg^2+^ with a congruent modulation of their known targets Runx2, Smad1, and Osterix, our results provide a potential mechanistic explanation of the observed upregulation of these master switches of osteogenesis during the course of VC.

## 1. Introduction

Vascular calcification (VC) is characterized by a pathological deposition of mineralized matrix in the vascular wall. VC is particularly associated with atherosclerosis, diabetes, and chronic kidney disease (CKD) and is rarely linked to genetic mutations [1, 2]. Clinically, VC is reflected in changes in parameters such as pulse pressure, coronary artery calcification, intima/media thickness, or pulse wave velocity [3]. The presence of VC leads to increased mortality rates in patients with CKD compared to the general population, due to increased intimal and medial calcifications of the large arteries [4]. VC is now described as a tightly regulated process sharing similarities with bone formation [5]. This active process involves the alteration of the contractile phenotype of vascular smooth muscle cells (VSMC) by specific exogenous stimulation. Indeed, exposure of VSMC to high phosphate and/or high calcium concentrations leads to an increase in mineralization, implying pathways involved in osteogenesis [5]. Furthermore, the specific upregulation of transcription factors such as Core-binding factor 1 *α* (Cbfa1)/Runt-related transcription factor 2 (Runx2), osterix (Osx), or transcription activators like Smad proteins are major features of both osteogenesis and VC [6].

Over the last decade, a novel class of regulators emerged as repressors of gene expression. Constituted of 18 to 25 nucleotides, microRNAs (miRs) are small, noncoding, regulatory RNAs. In the current canonical model [7], they act either by inducing an inhibition of the translation of their target mRNAs or via their degradation. A single miR is able to regulate the expression of multiple genes because of its ability to bind to its mRNA targets as either a perfect or imperfect complement. The human genome may encode more than 1 500 miRs that could target about 30% to 60% of the genes expressed in the various human cell types. Conversely, studies soon showed correlations between miRs expression and diseases and miRs have recently entered the cardiovascular field [8]. To focus more precisely on the mechanisms that cause VC, reports at first implicated miRs during the VSMC phenotypic switch and the vascular remodeling [9, 10]. Concomitantly, miR signatures were found during osteoblast and osteoclast differentiation [11–14], implying miRs which were later involved in VSMC-driven VC [15–18].

Magnesium (Mg^2+^) has recently been introduced as a new player in the field of VC. Since an inverse relationship between serum Mg^2+^ concentrations and VC was reported [19], a limited number of clinical as well as* in vitro* studies assessed a potential beneficial effect of Mg^2+^ reviewed in [20]. During the last four years, several studies confirmed that Mg^2+^ supplementation alleviates VC in both rodent and bovine models [21, 22]. Two studies further detailed the mechanistic aspects involved in phosphate-induced VC of human aortic VSMC (HAVSMC) [23, 24]. The various studies showed that Mg^2+^ negatively regulates VC through transient receptor potential melastatin (TRPM)7 activity and modulates expression of calcification markers such as anticalcification proteins (Osteopontin, Matrix Gla Protein), osteogenic proteins (Osteocalcin, Bone Morphogenetic Proteins), and osteogenic and VC related transcription factors (Cbfa1/Runx2 and Osx).

In the present study, we investigated a panel of miRs during Mg^2+^ attenuated inorganic phosphate- (Pi-) induced VC (Table 1).

This selection of miRs was extrapolated from the literature, which was sparse at the initiation of the study, as well as miRNA databases. We chose miR-29b, -30b, -125b, -133a, -143, -204, and -223 because they were shown to play a key role in the course of VSMC phenotypic switch, osteogenesis, or VC. Additionally, after browsing miR databases, we selected miRs that could target key mediators of VC such as col1a1, cbfa1/Runx2, Smad1, and Osx. For the first time in a human model, we were able to show the role of several miRs in the course of VC and thus gain additional mechanistic insights into the mode of action and beneficial effects of Mg^2+^ during this process.

## 2. Materials and Methods

### 2.1. Chemicals

All chemicals were purchased from Sigma unless otherwise stated.

### 2.2. Cell Culture of HAVSMC

Primary human VASMC were isolated in our laboratory from explants of human aortic tissue obtained with appropriate ethical approval. The samples were obtained after aortic valve bypass surgery or other types of surgery on the aorta from patients with various cardiovascular diseases (Pr Caus, Pôle Coeur Thorax Vaisseaux, CHU Amiens, France). This protocol was approved by the French Ethics Committee “Comité de Protection des Personnes (CPP) Nord-Ouest II” under ID #2009/19. The investigations were performed according to the principles outlined in the Declaration of Helsinki for use of human tissue or subjects. The medial tissue was separated from segment of human aorta after removal of endothelium. Small pieces of tissue (1-2 mm^2^) were placed in culture dish in Dulbecco's Modified Eagle's Medium (DMEM) supplemented with 15% of fetal bovine serum (FBS, Dominique Dutscher), 4.5 g of glucose, 1 mmol/L of pyruvate, 100 U/mL of penicillin, and 100 *μ*g/mL of streptomycin in a 5% CO_2_ incubator at 37°C. Cells that migrated from explants were collected when confluent. The cells were maintained in DMEM supplemented with 15% FBS, and the medium was replaced twice per week. HAVSMC were identified by their typical morphology, and purity of the primary cell culture was further checked by immunocytochemistry using a monoclonal antibody against the *α*-smooth muscle actin protein 1A4 (Acta2) (Santa Cruz Biotechnology) [25]. The cells were used between passages 6 and 12, during which time they were able to calcify. The cells isolated from 3 independent donors were used for the various experiments of the study.

For calcification assays, cells were seeded at 7 500 cells/well in 48-well plates and treated for the indicated times with various media conditions from 2 to 3 days after plating.

For RNA isolation, cells were seeded at 150 000 cells/well in 6-well plates. For the whole miR study, the experimental setup included conditions with or without 3 mM of Pi; magnesium was added to reach 1.5 or 2 mM. The samples were incubated and stopped at the time points 4 h, 24 h, day 3, day 7, and day 10. For all experiments, cells were treated as described in the calcification assays section.

### 2.3. Calcification Assays

DMEM medium initially contains 0.9 mM of Pi, 1.8 mM of Ca^2+^, and 0.8 mM of Mg^2+^. Calcification assays were conducted in 1% FBS DMEM, and Pi concentration was increased to reach 3 mM. The effect of Mg^2+^ on calcification was assessed at total concentrations of 1.5 or 2 mM. When indicated, the Pi and Mg^2+^ concentrations in the media were increased using NaH_2_PO_4_ and MgCl_2_ supplementation, respectively.

For precise calcium (Ca^2+^) measurements, cells were washed with PBS without Ca^2+^ and Mg^2+^ and then decalcified with 0.6 N HCl overnight. The calcium content was determined colorimetrically with the o-cresolphthalein complexone method (OCP). Briefly, the principle of this method is based on the purple colored complex formed by Ca^2+^ with o-cresolphthalein complexone in an alkaline medium. The optical density (OD) of the samples was measured with a spectrophotometer at 565 nm and compared to a curve calibrated with Ca^2+^ standards [26]. The protein content was measured using Bio-Rad protein assay reagent (Bio-Rad) according to the manufacturer's protocol. The Ca^2+^ content of the cell layer was normalized to protein content.

For alizarin red staining (AR), cells were washed with PBS and fixed with ethanol 95%. Then samples were exposed to alizarin red 40 mM (pH 4.2). After two washing steps, the wells were photographed to document mineralization.

For Von Kossa staining (VK), cells were fixed with ethanol 95% for 15 min and rinsed and incubated with 5% AgNO_3_ for 30 min. Cells were further rinsed with water, incubated for 5 min in a photographic developer solution, washed with water, then incubated for 5 min with a 5% sodium thiosulfate solution to remove unreacted silver, washed, and finally dried. Following their acquisition with a Photometrics CH250 CCD camera, pictures of wells were processed using the ImageJ software (NIH).

### 2.4. Specific Controls

Media from the various experimental conditions were assessed for correct Ca^2+^, Pi, and Mg^2+^ levels using an ADVIA 1800 Siemens autoanalyzer (Siemens Healthcare Diagnostics). No change in pH was observed at Pi 3 mM with or without addition of Mg^2+^. This excludes a potential role of medium acidification in the observed decrease of mineralization.

In previous experiments [24], we checked whether the effects of MgCl_2_ on calcification reduction were due to chloride ions in MgCl_2_ salt, but addition of 2.4 mM NaCl (the maximum concentration of Cl^−^ used) did not inhibit calcification after 10 and 14 days of culture in presence of 3 mM Pi using AR, VK, and OCP methods (data not shown).

### 2.5. RNA Isolation and Quantitative PCR

Total RNA from HAVSMC was isolated with the mirVana Isolation Kit (Applied Biosystems, Life Technologies) as per the manufacturer's instructions. Total RNA was further subjected to DNAse-I digestion (Ambion, Life Technologies). For selected miRs, samples of 50 ng of RNA were reverse-transcribed in a final volume of 25 *μ*L using the Applied Biosystems Taqman assay probes with the Taqman MicroRNA Reverse Transcription Kit according to the manufacturer's protocol. Real-time quantitative PCR (q-PCR) was run on a StepOnePlus system (Applied Biosystems) using Taqman assay probes. For selected mRNA targets (collagen 1A1, Runx2, Smad1, and Osx), samples of 50 ng of RNA were reverse-transcribed in a final volume of 25 *μ*L using a High-Capacity cDNA Synthesis Kit (Applied Biosystems) according to the manufacturer's protocol. Real-time q-PCR was then run on StepOnePlus using 10 *μ*M primers of the selected targets (Table 2) with the Power SYBR Green PCR Master Mix (Applied Biosystems).

The U6 small nuclear RNA and GAPDH mRNA were used as endogenous controls for miR and mRNA expression, respectively (for shown results). Alternatively, U48 small nuclear RNA and *β*-actin were also used as additional endogenous controls and yielded the same trend of results (data not shown).

### 2.6. miRNA Target Site Prediction

A search for predicted target miR was performed with the databases TargetScan (http://www.targetscan.org/), miRanda (http://www.miranda-im.org/), and PicTar (http://pictar.mdc-berlin.de/).

### 2.7. Statistical Analysis

All results are expressed as mean ± standard error of the mean (SEM). For calcification assays, statistical significance was determined by variance multigroup analysis (ANOVA) using the Fisher PLSD posttest. For Taqman and SYBR green q-PCR experiments, the nonparametric Mann-Whitney test was used because of the standard deviation observed between donors. A *p* value < 0.05 was considered to be significant.

## 3. Results

### 3.1. Mg^2+^ Reduced Pi-Induced Mineral Deposition in Tested Samples

**Figure 1:**
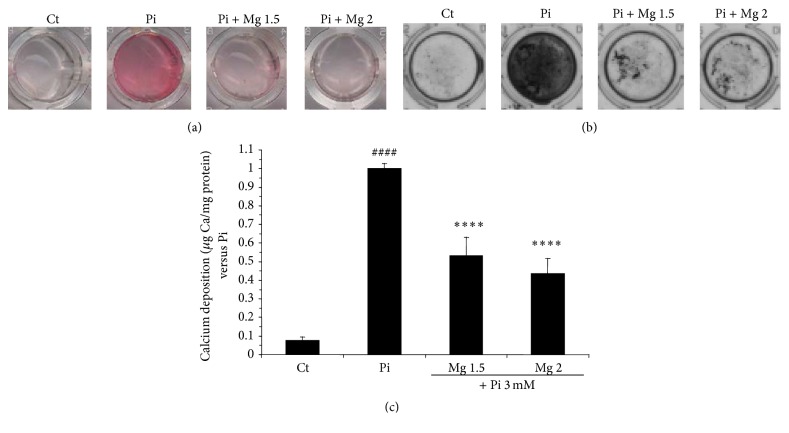
Magnesium prevents Pi-induced mineral deposition in HAVSMC. HAVSMC were cultured for 14 days at the indicated concentrations of Mg^2+^, 1.5 and 2 mM (Mg 1.5 and Mg 2, resp.), and in the presence of 3 mM Pi (Pi). (a) Alizarin red staining pictures, to allow visualization of calcium deposition, are representative of the respective conditions. (b) Von Kossa staining pictures, to allow visualization of calcium deposition, are representative of the respective conditions. (c) Calcium deposition from HAVSMC of the three different donors was assessed using OCP with increasing concentration of Mg^2+^ and induction of calcification by Pi. Data are represented as a ratio of Pi and are expressed as mean ± SEM for each condition (*n* = 12). Pi 3 mM condition was set to 1. Statistics were realised with ANOVA multigroup analysis using Fisher PSLD posttest. The control condition (Ct) is significantly different from all conditions. ^####^ *p* < 0.0001 versus Ct and ^*∗∗∗∗*^ *p* < 0.0001 versus Pi.

As previously described [24], the deposition of calcified matrix occurs by rising Pi concentration up to 3 mM. As shown in Figure 1(c), Mg^2+^ significantly decreases the amount of Ca^2+^ quantified by the OCP method after 14 days of incubation with the indicated conditions. These samples were concomitantly assessed for miR and mRNA expression. Here, AR and VK staining were only performed as qualitative tests to confirm the occurrence of the calcium phosphate deposition in our samples (Figures 1(a) and 1(b)). Calcification was checked at day 14 because we previously found that this was the optimal time point to show the efficiency of Mg^2+^ to prevent VC [24].

### 3.2. miR-125b and miR-223 Are Not Significantly Regulated during the Course of Calcification

miR-125b expression was very stable and remained unchanged in all of the tested conditions over the time course (4 h, 24 h, day 3, day 7, and day 10) for all donors (data not shown). This result seems to be in accordance with a previous work showing that miR-125b was not significantly downregulated until day 21 under calcifying conditions [15]. In this work, the authors did not test calcification time points prior to 7 days for miR-125b regulation. Here, we demonstrate that miR-125b is regulated by Pi neither at earlier time points (4 h, 24 h, and day 3) nor at day 7 or 10 as already mentioned in [15]. Likewise, Mg^2+^ had no effect on miR-125b expression (data not shown). In a different way, miR-223 was not found to be significantly regulated in the tested conditions during the whole time course, partly due to a high variability of its expression among the donors. Anyway, Mg^2+^ did not seem to have a beneficial effect in restoring basal miR-223 expression. Thus, the data suggest that the regulation of this miR cannot explain the beneficial effect of Mg^2+^ in attenuating VC (data not shown).

### 3.3. miR-29b and miR-204 Expression Is Markedly but Not Significantly Altered

**Figure 2:**
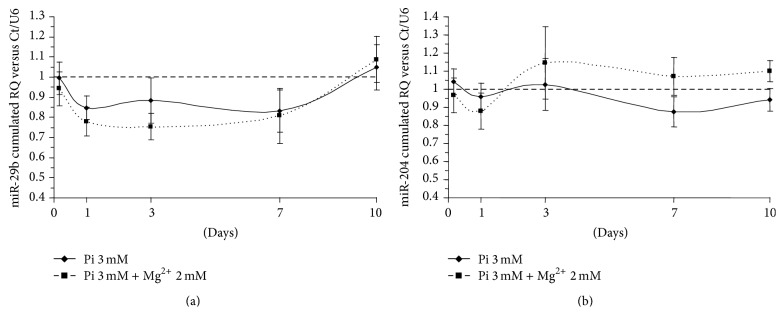
miR-29b and miR-204 expression is not significantly altered. Pi-induced HAVSMC were assessed at the time points 4 h, 24 h, day 3, day 7, and day 10. miR-29b and miR-204 were quantified using TaqMan microRNA assays (Applied Biosystem) on total RNA by q-PCR analysis and compared to control cells treated with basal medium. The control condition was set to 1 (dashed line). The cumulated relative quantity (RQ) of the three different donors of HAVSMC is represented as a ratio of the control condition and is expressed as mean ± SEM for each condition (*n* = 6). U6 small nuclear RNA was used as an internal reference gene. Statistics were realised with the Mann-Whitney test. No statistical significance was found.

The regulation profile of miR-29b shows a marked tendency to decrease in the presence of Pi (3 mM), particularly until day 7. However, the statistical significance was not reached due to the variability observed between the tested donors, meaning that the SEM is large compared to the observed variation. Adding Mg^2+^ (2 mM) in the calcifying condition (Pi 3 mM) was not effective to recover or tend to return to the basal expression of miR-29b. This is shown by the expression curves which progress tightly together (Figure 2(a)).

In Figure 2(b), the expression levels of miR-204 stay within the control range at days 1 and 3 and slightly decrease from day 7 onwards in the presence of Pi 3 mM. Conversely, miR-204 was upregulated from days 3 to 10 when Mg^2+^ (2 mM) was added to the calcifying condition. However, no significance was found between the conditions and time points because of the substantial interdonor biological variations.

### 3.4. Mg^2+^ Is Able to Reverse a Pi-Induced Decrease in miR-30b Expression

**Figure 3:**
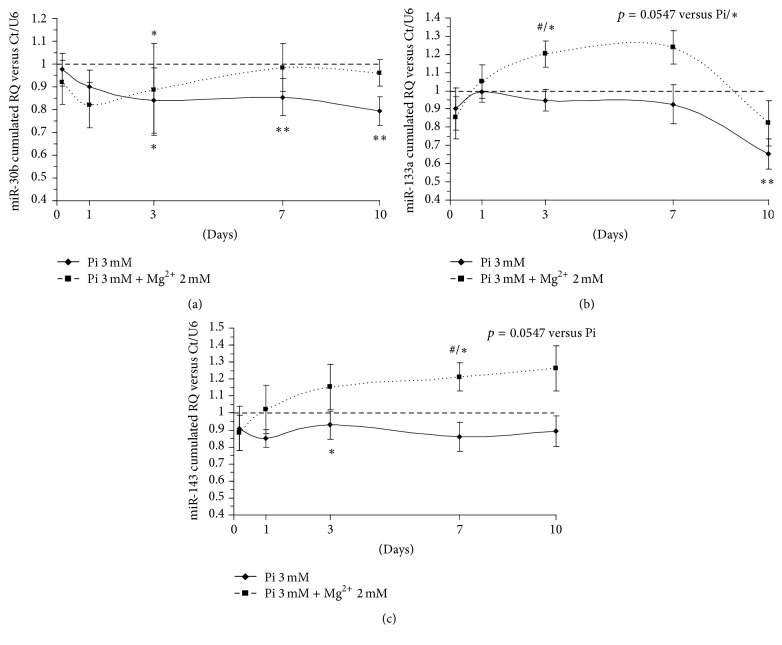
miR-30b, miR-133a, and miR-143 expression is deregulated by Pi and restored by Mg^2+^. Pi-induced HAVSMC were assessed at the time points 4 h, 24 h, day 3, day 7, and day 10. miR-30b (a), miR-133a (b), and miR-143 (c) were quantified using TaqMan microRNA assays (Applied Biosystem) on total RNA by q-PCR analysis and compared to control cells treated with basal medium. The control condition was set to 1 (dashed line). The cumulated relative quantity (RQ) of the three different donors of HAVSMC is represented as a ratio of the control condition and expressed as mean ± SEM for each condition (*n* = 6). U6 small nuclear RNA was used as an internal reference gene. Statistics were realised with the Mann-Whitney test. ^*∗*^ *p* < 0.05 versus Ct, ^*∗∗*^ *p* < 0.01 versus Ct, and ^#^ *p* < 0.05 versus Pi.

Data on miR-30b regulation show a significant, progressive decrease in its expression in the calcifying condition from day 3 onwards. This decrease is worsened throughout day 10, reaching higher statistical significance. Adding Mg^2+^ to the calcifying condition resulted in an initial decrease of miR-30b expression that quickly rose and then returned to the basal expression level from day 7 onwards, being nonsignificantly different from the control condition set to 1 (Figure 3(a)). The results are in line with previous publications suggesting that the miR-30b decrease was a procalcifying event [18] in human smooth muscle cells.

### 3.5. Mg^2+^ Potentiates miR-133a Expression and Prevents Its Pi-Induced Decrease

Regarding the time course of miR-133a regulation for the various conditions, HAVSMC cultured with 3 mM Pi led to a progressive decrease of miR-133a expression, whereas the addition of Mg^2+^ resulted in an upregulation of miR-133a (Figure 3(b)). Beginning at day 3, statistical significance is found between the Pi 3 mM and Pi 3 mM + Mg^2+^ 2 mM conditions as well as between control (set to 1, dashed line) and Pi 3 mM + Mg^2+^ 2 mM. Similar results were found at day 7. After 10 days of induced calcification, miR-133a expression levels were lowered in conditions containing Pi. However, only strong statistical significance was found between control and Pi 3 mM conditions. Thus, the addition of Mg^2+^ 2 mM only partially reverses the decrease of miR-133a. The downregulation of miR-133a is consistent with findings in [11] in which miR-133/135 were downregulated during osteogenic differentiation.

### 3.6. Mg^2+^ Prevents miR-143 Pi-Induced Decrease Expression

Figure 3(c) shows the time course of miR-143 regulation for the various conditions. At day 3, Pi 3 mM significantly lowered miR-143 expression, and this decrease remained stable until day 10, although not reaching statistical significance on days 7 and 10. Conversely, Mg^2+^ constantly increased miR-143 levels from the early time points until day 10 over the time course, resulting in a significant difference between the Pi 3 mM and Pi 3 mM + Mg^2+^ 2 mM conditions on day 7. A decrease of miR-143/145 is correlated with several important cardiovascular diseases; for review see [9]. Downregulation of miR-143/145 was previously studied during HAVSMC Pi-induced calcification by our team [27], thus supporting the present results.

### 3.7. Expression of Collagen 1A1 and Runx2 Is Not Significantly Upregulated by Pi

**Figure 4:**
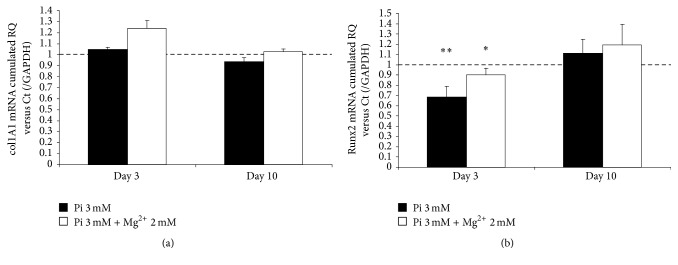
The expression of col1A1 and Runx2 mRNA is not markedly induced by Pi. Pi-induced HAVSMC were assessed at day 3 and day 10. Total RNA was extracted and reverse-transcribed, and expression of col1A1 (a) and Runx2 (b) was analyzed by q-PCR and compared to control cells treated with basal medium. The control condition was set to 1 (dashed line). The cumulated relative quantity (RQ) of the three different donors of HAVSMC is represented as a ratio of the control condition and expressed as mean ± SEM for each condition (*n* = 6). GAPDH was used as an internal reference gene. Statistics were realised with the Mann-Whitney test. ^*∗*^ *p* < 0.05 versus Ct and ^*∗∗*^ *p* < 0.01 versus Ct.

We chose to present the data concerning miR specific targets at day 3 and day 10 for all the selected targets because the most marked miR modulations are observed between these two time points. As indicated in Table 1, miR-29b targets collagen 1A1 (col1A1) mRNA, a classic marker of calcification. The expression of collagen 1A1 was not significantly modulated in our experimental setup (Figure 4(a)). This result could be related to the lack of a significant change in miR-29b expression.

The expression of Runx2 mRNA is regulated by several miRs: miR-30b, miR-133a, and miR-204 (Table 1). We mentioned that miR-30b and -133a were found to be differentially expressed (Figures 3(a) and 3(b)). Corresponding Runx2 regulations were not observed. Surprisingly, Runx2 was found to be significantly downregulated at day 3, in both the presence and absence of Mg^2+^, whereas at day 10 expression rose to lie nonsignificantly above control levels (Figure 4(b)). We could hypothesize that the rise in Runx2 expression might continue after day 10 for the following reasons. The Pi-induced downregulation of miR-30b and -133a is at its maximum at day 10. The effects of miR downregulation, however, are not seen concomitantly on the target expression. A further time point is needed at least 24 h to several days later to detect the maximum effects of miR expression on their relative mRNA targets. This suggests that the experimental setup was probably concluded too early and missed the time window that would detect a significant upregulation.

### 3.8. Upregulation of Smad1 and Osx Expression Is Restored by Mg^2+^

**Figure 5:**
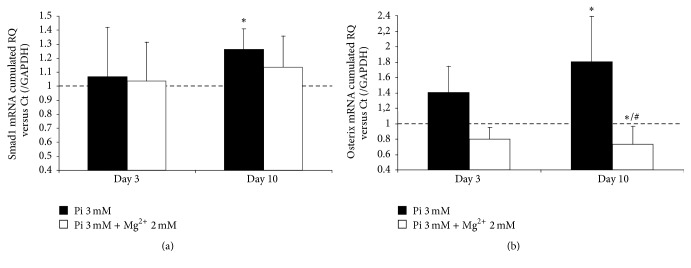
The expression of Smad1 and Osterix mRNA is increased by Pi induction whereas the presence of Mg^2+^ prevents an upregulation. Pi-induced HAVSMC were assessed at day 3 and day 10. Total RNA was extracted and reverse-transcribed, and expression of Smad1 (a) and Osterix (b) was analyzed by q-PCR and compared to control cells treated with basal medium. The control condition was set to 1 (dashed line). The cumulated relative quantity (RQ) of the three different donors of HAVSMC is represented as a ratio of the control condition and is expressed as mean ± SEM for each condition (*n* = 6). GAPDH was used as an internal reference gene. Statistics were realised with the Mann-Whitney test. ^*∗*^ *p* < 0.05 versus Ct and ^#^ *p* < 0.05 versus Pi.

In a recently published study [28], a link was established between miR-30b regulation and mRNA expression of Smad1, Runx2, and caspase-3 in human primary aortic valve interstitial cells. These three mRNA were identified and confirmed as potential target genes of miR-30b (Table 1). Here, we assessed the Smad1 expression in cultured HAVSMC and found a marked and significant increase of Smad1 at day 10 in the calcifying condition (Figure 5). Conversely, in the presence of Mg^2+^, Smad1 expression is decreased towards control level. This correlates with the observed miR-30b regulation.

A recent report established Osx as a target of miR-143 in* in vitro* osteogenesis [29]. In this work, the most prominent modulation was observed for Osx mRNA expression. As shown in Figure 5(b), Osx expression was markedly but not significantly upregulated at day 3 in the calcifying condition (by approx. 40%) and further and significantly upregulated at day 10 (by approx. 80%). The presence of Mg^2+^ abrogated the Osx upregulation, at both day 3 and day 10. Moreover, the addition of Mg^2+^ resulted in a significant decrease of Osx expression at day 10 compared to the control and the calcifying condition (Pi 3 mM). As suggested in [29], these results are correlated and could be mirrored to miR-143 expression.

## 4. Discussion

The present study performed a limited miR screening based on available literature (implications in VSMC phenotypic switch, osteogenesis, or VC, Table 1) in HAVSMC primary cells. The aim was to detect miRs implicated in VC, regulated by Mg^2+^, and subsequently to study the targets of the most relevant miRs. It reveals three main findings: (i) our screening showed a downregulation of key miRs such as miR-30b, miR-133a, and miR-143 during Pi-induced calcification of HAVSMC; (ii) osteogenesis and VC markers related to these miRs, such as Smad1 and Osterix, were found to be modulated accordingly; and (iii) Mg^2+^ had a protective effect by interfering with the Pi-induced VC process as the modulations of the affected miRs and their related targets were partially abrogated or even improved.

Based on literature and confirmed by miR databases, a selection of 7 miRs was assessed in our HAVSMC model during Pi-induced calcification at 4 h, 24 h, day 3, day 7, and day 10. Our results confirmed the implication of miR-30b in calcification and brought miR-133a as well as miR-143 from phenotypic switch and vascular remodeling into the field of VC. To briefly comment on the results of the other selected miRs, Goettsch et al. [15] found a statistically significant increase of miR-125b in human coronary artery smooth muscle cells after 21 days in calcifying conditions (10 mM *β*-glycerol phosphate/10 nM dexamethasone/100 *μ*M ascorbate phosphate). In our model (HAVSMC), miR-125b expression was found to be stable for 10 days in calcifying conditions (3 mM Pi), confirming a late involvement of miR-125b in the VC process. Furthermore, the high variability among the tested donors might have prevented us from obtaining statistical significance in the Pi-induced regulation of miR-29b, miR-204, and miR-223. It seems that the rather limited number of samples could be a limitation of our study. Adding several other donors could be an option to improve the significance and clarify the involvement of the selected miRs in VC. For miR-29b, the Pi-induced expression profile resembles that presented in [13], where miR-29b was first decreased before being increased in MC3T3-E1 and rat calvaria osteoblast models of osteogenesis. For miR-204, the overall expression profile in the calcifying condition showed a constant but not significant decrease from day 3 until day 10. This decrease is similar to what was described in primary mouse aortic SMC in [17], where the expression of miR-204 constantly decreased from day 3 until day 14. For both miR-29b and miR-204, further investigations should be conducted in HAVSMC or in other human vascular models to clarify the role that these miRs as well as their putative targets play in the VC process.

Before the initiation of our study, Balderman et al. reported a decrease of miR-30b/c during BMP-2 (Bone Morphogenetic Protein-2) induced VC of human artery smooth muscle cells [18]. Knockdown of miR-30b/c established Runx2 as their preferential target during VC. Of note, Smad proteins (1/5/8) were activated by BMP-2 during the VC process. Moreover, a siRNA strategy to decrease Smad1 mRNA and consequently Smad1 protein levels prevented the decrease of miR-30b/c. To date, the whole miR-30 family has been implicated in the osteogenesis process [30]. In our experimental setup, miR-30b was found to be downregulated during Pi-induced VC, confirming previous findings. This decrease was abolished by the addition of Mg^2+^ to the calcifying medium. In the meantime, miR-30b was found to be a multifunctional regulator of aortic valve interstitial cells in calcified aortic valve disease [28]. Indeed, its levels were decreased during calcification, whereas its modulation altered Runx2, Smad1, phosphorylated Smad1/5/8, and caspase-3 protein levels; that is, target levels were increased by miR-30b inhibition and decreased by overexpression strategies.

The implication of miR-133a in a mineralization process was first described during BMP-2 induced osteoblastogenesis [11], where it was found to be downregulated. A sequence analysis indicated the presence of a putative miR-133a binding site located in the 3′ UTR of Runx2 mRNA. Inhibition and overexpression of Runx2 protein as well as other calcification markers were shown using ectopic miR-133a and anti-miR-133a, respectively. It is of note that, despite the use of ectopic anti-miR-133a, Runx2 mRNA levels remained unaffected. Lately, miR-133a was shown to be downregulated in primary murine vascular SMC calcified by the addition of 10 mM *β*-glycerol phosphate [31]. Thus, our data expand Liao et al. observations on miR-133a downregulation during VC to a human vascular model. Conversely, upregulation of miR-133a was found when Mg^2+^ was added to the calcifying condition.

Considering the previous studies, we decided to assess Runx2 and Smad1 mRNA expression to see the potential consequences of a miR-30b and miR-133a Pi-induced decrease. Unfortunately, we were not able to provide evidence of a significant rise in Runx2 mRNA levels, as de Oca et al. did, using a higher Pi concentration than us during similar incubation times in HAVSMC [23]. Indeed, convincingly showing significant upregulation of Runx2 mRNA expression appears to be a challenge in human VC while browsing at the literature. A main finding of our study was the demonstration of the significant Pi-induced mRNA upregulation of Smad1, another major player in the field of VC. It is now clearly established that cooperative interactions between Runx2 and BMP/Smad signaling pathways occur to induce the osteoblast phenotype [32]. Moreover, a physical interaction between Runx2 and Smad1/3/5 appears essential for osteogenic activity* in vitro* [33]. As similarly found in human valve interstitial cells [28], an increase of Smad1 mRNA expression is reported during VC in our study.

First described to contribute to the VSMC phenotypic switch [18, 27], miR-143 was recently involved in VC of uremic mice [34]. Indeed, vascular smooth muscle-specific miR-143 expression was decreased in aortas of ApoE^−/−^ mice in states of atherosclerosis and/or CKD during VC progression. During osteogenic differentiation of MC3T3-E1 cells, miR-143 had decreased levels of expression and had a role in osteogenesis inhibition. Moreover, Osx was identified to be a direct target of miR-143 [29]. Here, regarding miR-143 modulation, Osx was tested as its most important putative target in the field of VC. A prominent increase in the Osx mRNA level was found in HAVSMC after 10 days in calcifying conditions. Osx is characterized as a necessary and essential actor in mineralizing tissues [29, 32]. Parallel observations in animal models demonstrated that while Osx-knockout mice expressed wild-type Runx2 levels in osteoblasts, Osx is not expressed in Runx2-knockout mice, suggesting that Osx acts downstream of Runx2 [35]. Alternatively, additional Runx2-independent or synergistic pathways were involved [36, 37]. Recently, the functional and physical interactions between Osx and Runx2 through their respective phosphorylations by MAPK were shown to modulate the transcriptional osteogenic program [37]. Both Runx2 and Osx are able to bind to their responsive sequences on the promoters and interact with each other via regulatory regions that lead to stabilization of the transcriptional complex. In HAVSMC, de Oca et al. showed a slight and nonsignificant increase of Osx mRNA expression during VC [23]. Our results are now definitely correlating the upregulation of Osx mRNA to the Pi-induced VC in HAVSMC. Such an observation underlines the critical role of Osx in HAVSMC Pi-induced VC as part of the process in a Runx2-independent or synergistic manner.

**Figure 6:**
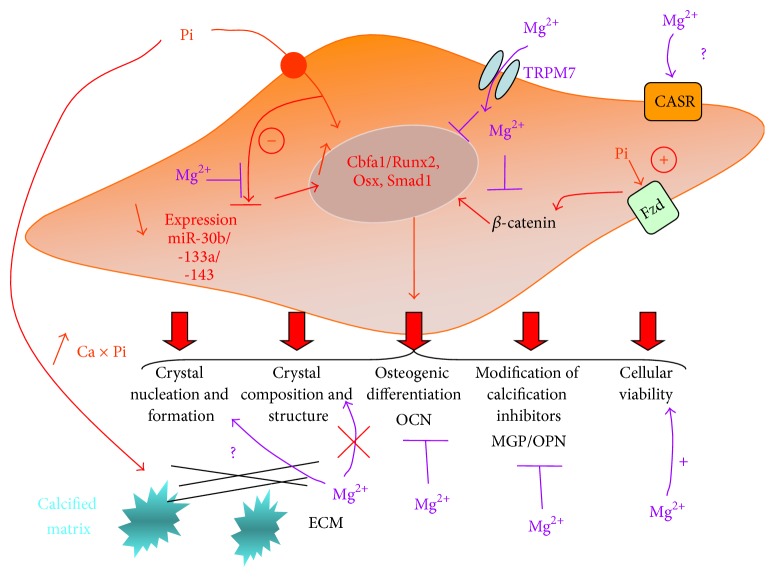
Multiple modes of action of Mg^2+^ in attenuating Pi-induced VC in HAVSMC. This figure summarizes all the knowledge from the previous [23, 24, 38] and the current report concerning Mg^2+^ from its entry into the cell through TRPM7 and its role in crystal composition and structure, modifications of calcification inhibitors, cell viability, osteogenic differentiation, inhibition of the Wnt/*β*-catenin pathway, and finally the influence of Mg^2+^ on osteogenes expression (Runx2, Osterix, and Smad1) through specific miR modulation (miR-30b, miR-133a, and miR-143). The question marks indicate undetermined mechanisms. ECM, extracellular matrix; OCN, osteocalcin; MGP, Matrix Gla Protein; OPN, Osteopontin; Ca × Pi, calcium by phosphate product; TRPM7, transient receptor potential melastatin-7; Fzd, Frizzled-3 receptor (Wnt signaling proteins); CASR, calcium sensing receptor.

Magnesium was tested in this study and results confirmed its beneficial role in preventing the VC process. Although previous studies already established the beneficial role of Mg^2+^ and demonstrated its ability to counteract the main calcification mediators [21–24, 38], the precise intracellular mode of action of Mg^2+^ remains elusive. Our data suggest that Mg^2+^ is able to antagonize the Pi-induced decrease of 3 miRs (miR-30b, miR-133a, and miR-143) involved in mineralization processes or SMC phenotypic switch. In HAVSMC, the decrease in these three miRs is a potential signature indicating that SMC are engaged in VC process. We hypothesize that the addition of Mg^2+^ abrogates the required inhibition of these miRs thus suppressing overexpression of two key factors of VC, Runx2 and Osx. These insights complete previous publications studying Pi-induced VC in HAVSMC [23, 24, 38]. The past and present findings are summarized in Figure 6, where the multiple modes of action of Mg^2+^ in Pi-induced VC are depicted: from its entry into the cell through TRPM7, its modulation of Ca/P crystal composition and structure, its modifications of calcification inhibitors, enhancement of cell viability, suppression of osteogenic differentiation, inhibition of Wnt/*β*-catenin pathway, and finally its active influence on osteogenesis (Runx2, Osx, and Smad1) through specific miR modulation (miR-30b, miR-133a, and miR-143).* In vitro*, magnesium's influence in Pi-induced VC could be tested in additional works on initiation of the crystal formation or on a putative activation of the calcium sensing receptor (CaSR).

In summary, we found that Pi, the most prominent natural inducer of VC, was able to decrease the expression of miR-30b/miR-133a/miR-143. These modulations represent a phenotypical switch and could be seen as a signature indicating that VC is initiated in HAVSMC and, by extension, into the arterial wall. The respective mRNA targets, Osx and Smad1, were modified accordingly towards osteogenic induction. The addition of Mg^2+^ was able to restore basal levels or even to upregulate miR expression. We are now able to assert that Mg^2+^ is effective relatively early during Pi-induced VC by cancelling osteogenic gene expression through miR-30b/miR-133a/miR-143 expression reinforcement, resulting in a retention of the SMC phenotype. How Mg^2+^ selectively modulates the 3 miRs is not yet known and could be a matter of additional investigations. To date, only nonspecific roles could be attributed to Mg^2+^ in miR maturation [39–41]. Obviously, the relevance of our past and present findings on the influence of magnesium during VC needs to be confirmed in a cohort of CKD patients in a clinical setting in further works. The use of magnesium as a drug, to lower serum calcium and phosphorus, and its effect on outcomes in CKD patients was detailed in [20]. To our knowledge, magnesium-containing phosphate binders have not yet been investigated for quantitative VC reduction in a controlled, prospective clinical setting, and this step now appears necessary.

## Figures and Tables

**Table 1:** Selection of specific miRs, involved in VSMC phenotypic switch, osteogenesis, or VC, in different cell types, and their effects in cell physiology and related targets as reported in the literature.

miRs	Regulation	Cell type & physiology	Origin	Targets	Reference
29b	Increased	MC3T3 preosteoblastic cells, osteogenesis	Mouse	Collagen 1A1	[13]
30b/30c	Decreased	Coronary artery smooth muscle cells, calcification	Human	Runx2	[18]
30a/30b/30c/30d/30e	Decreased (30b/30c) Increased (30a/30d/30e)	Mesenchymal stem cells/bone marrow stromal cells/MC3T3-E1, osteogenesis	Mouse	Runx2, sox9, osteopontin, and various others	[30]
125b	Decreased	Coronary artery smooth muscle cells	Human	Osterix (sp7)	[15]
133a/135a	Decreased	C2C12 cells, osteogenesis	Mouse	Runx2 (miR-133a)/Smad5 (miR-135a)	[11]
133a	Decreased	Aortic smooth muscle cells and isolated arteries, phenotypic switch, and vascular remodeling	Rat	Acta2, sm-myosin heavy chain	[10]
143/145	Increased	Pulmonary artery smooth muscle cells, phenotype modulation	Human	klf4/myocardin	[9]
143/145	Decreased	Aortic smooth muscle cells, migration and phenotypic switch	Human	klf4/myocardin	[9]
204	Decreased	Aortic smooth muscle cells, calcification	Mouse	Runx2	[17]
223	Increased	Aortic smooth muscle cells, migration and phenotypic switch	Human	rhoB/mef2c/Acta2	[27]

**Table 2:** Primers used for q-PCR were published previously or designed through the use of PrimerBank [30, 31, 42].

Gene name	Forward (5′-3′)	Reverse (5′-3′)	GenBank
GAPDH	ATGGAAATCCCATCACCATCTT	CGCCCCACTTGATTTTGG	NM_002046
*β*-actin	CCTCACCCTGAAGTACCCCA	TGCCAGATTTTCTCCATGTCC	NM_001101
Collagen 1A1	ACGAAGACATCCCACCAATCAC	TCATCGCACAACACCTTGC	NM_000088
Osterix	CCTCTGCGGGACTCAACAAC	AGCCCATTAGTGCTTGTAAAGG	NM_152860
Runx2	AGCTTCTGTCTGTGCCTTCTGG	GGAGTAGAGAGGCAAGAGTTT	NM_001024630
Smad1	TTCCATGCCTCCTCCACAAG	AGGCATTCGGCATACACCTC	NM_005900
